# Surgical Indications and Clinical Results of Patients with Exchanged Intraocular Lenses in a Tertiary Eye Hospital

**DOI:** 10.4274/tjo.69379

**Published:** 2016-08-15

**Authors:** Sevim Kavuncu, Aslıhan Esra Omay, Mehmet Hakan Tırhış, Pelin Yılmazbaş

**Affiliations:** 1 Ulucanlar Eye Training and Research Hospital, Ankara, Turkey

**Keywords:** Bullous keratopathy, intraocular lens exchange, intraocular lens subluxation, pseudoexfoliation

## Abstract

**Objectives:**

To evaluate the demographics, surgical indications and clinical results of patients with repositioned or explanted intraocular lens (IOL) in a tertiary referral eye hospital.

**Materials and Methods:**

Forty-eight eyes of 48 patients that underwent surgery to exchange or reposition the IOL at Ulucanlar Eye Training and Research Hospital between 2009 and 2013 were included in the study. Medical records of patients were evaluated for surgical indications, time elapsed since initial operation, preoperative and postoperative best corrected distance visual acuity and the presence of ocular disease.

**Results:**

The mean age of the 31 male and 17 female patients was 64.91±14.26 years. Median time between the initial and final operations was 36.0 months. Pseudoexfoliation syndrome (PEX) was present in 25% of the patients. There was history of previous vitreoretinal surgery in 18.8% of patients, ocular trauma in 6.3%, high myopia and refractive surgery in 4% of patients. In the first operation the IOL was implanted in the sulcus in 50%, in the bag in 27.1%, and in the anterior chamber in 20.8%; following the final surgery the IOL was in the sulcus in 27.1%, in the anterior chamber in 22.9%, and fixated to the sclera in 10.4% of the patients, while the remaining 29.1% remained aphakic. Indication for the secondary surgery was IOL dislocation in 58%, corneal decompensation in 20.8% and IOL degeneration in 6.3%. In the final surgery, IOL was exchanged in 54.2% of the cases, removed in 31.3% of cases, and repositioned in 14.6%. Visual acuity improved by 1-3 lines in 52.3% and remained stable in 13.6% of the patients postoperatively.

**Conclusion:**

IOL exchange may be necessary at any time following cataract surgery due to surgical complications, IOL dislocation, biometric measurement errors and corneal decompensation. Factors such as vitreoretinal surgery and the existence of PEX increase the risk of IOL exchange surgery.

## INTRODUCTION

Phacoemulsification is the most commonly performed cataract surgery technique both in Turkey and abroad, and posterior capsule perforation is one the most feared complications. Inadequate posterior capsule support may lead to decentration of the implanted lens and result in complaints like vision loss, blurred vision and double vision. In cases with severe visual disturbances, removing the intraocular lens (IOL) may be necessary. Other than inadequate posterior capsule support, other potential reasons an IOL may need to be removed or exchanged postoperatively include incorrect refractive power of the IOL, visual disturbances (e.g. glare) caused by the IOL, and elevated intraocular pressure related to the IOL. Whether or not to replace a removed lens and the anatomic location of the new lens are decided based on surgeon experience, patient age, the condition of the posterior capsule, and the patient’s other ocular pathologies (glaucoma, diabetic retinopathy, asteroid hyalosis, low endothelial cell count, increased corneal thickness).^1,2^

In this study, we aimed to evaluate the demographic characteristics, indications for IOL removal and visual outcomes in patients undergoing IOL exchange or repositioning in a tertiary referral hospital.

## MATERIALS AND METHODS

The records of 86 patients who underwent IOL exchange surgery in Ulucanlar Eye Hospital between 2011 and 2014 were analyzed retrospectively. Forty-eight eyes of 48 patients who were followed for at least 6 months after the second surgery were included in the study. The patients’ age, gender, history of systemic disease, any additional ocular pathology, indication for IOL exchange, time elapsed since implantation of the first IOL, and the anatomic positions of the first and second IOLs were recorded. Patients with intravitreal dislocation of the IOL were excluded. Calculations using biometric measurements for both the sulcus and the bag were done to determine the power of the IOL to be implanted. Biometric evaluation of the fellow eye was used for patients with corneal opacity or for whom keratometric measurement could not be performed. Intracameral, subTenon’s or subconjunctival anesthesia was used during surgery. Approval for the study was granted by the Ankara Numune Training and Research Hospital Ethics Committee. Data were statistically analyzed as numerical values and percentages.

## RESULTS

The 48 patients in the study included 31 men and 17 women with a mean age of 64.91±14.26 (range, 26-87) years. The median time between the first and second surgeries was 36.0 months (range, 1-260 months).

Surgical indications were dislocated IOL in 58% (n=28), corneal decompensation in 20.8% (n=10), and IOL degeneration in 6.3% (n=3) of the patients (Table 1).

Twenty-five percent (n=12) of the patients were positive for pseudoexfoliation syndrome (PEX), 18.8% (n=9) had previous vitreoretinal surgery, 6.3% (n=3) had a history of iridophacodonesis or ocular trauma, and 4% (n=2) had high myopia or previous refractive surgery (Table 2). Clinical indications for previous vitreoretinal surgery was macular hole in 2 cases, diabetic retinopathy in 3, intravitreal hemorrhage in 2, endophthalmitis in 1 and choroidal neovascular membrane in 1 case. After the second surgery, 4 of the 13 PEX patients remained aphakic, while the IOL was implanted in the anterior chamber in 5 patients, in the sulcus in 2 patients and was fixated to the sclera in 2 patients. Eight of the patients in this study underwent their first surgery in our hospital; the remaining were operated at different times in other centers.

The IOL was exchanged in 54.2% (n=26) of the patients, removed in 31.3% (n=15) and repositioned in 14.6% (n=7) (Table 3). Initial IOL implantation was in the sulcus for 50%, in-the-bag for 27.1%, and anterior chamber for 20.8% of the patients; in the second surgery, IOL position was in the sulcus for 27.1%, anterior chamber for 22.9%, scleral fixation for 10.4%, and 14 patients (29.1%) remained aphakic (Table 4). Seven of the 14 patients who remained aphakic after the second surgery had bullous keratopathy; these patients’ initial IOLs had been implanted in the anterior chamber. These patients were 36, 26, 44 and 49 years of age. The IOL was removed due to corneal decompensation in 10 (60%) of the aphakic patients. For the remaining aphakic patients, there was insufficient posterior capsule support and the IOL could not be implanted in the anterior chamber due to uveitis (1 patient), glaucoma (2 patients), and iris defect (1 patient). Three of the 10 patients with corneal decompensation were under 55 years old and were scheduled for keratoplasty. The other 7 patients had fellow eyes within normal limits and did not accept the possible risks of keratoplasty; they underwent medical treatment and were followed in the cornea unit.

Change in visual acuity after the second surgery was a loss of 4 or more rows in 6.8%, loss of 1-3 rows in 22.7%, gain of 1-3 rows in 52.3% and gain of 4 or more rows in 4.5% of the patients; visual acuity remained the same in 13.6% of the patients (Table 5).

## DISCUSSION

With the major technological advancements made in cataract surgery, indications for postoperative IOL exchange have changed over time. Analysis of studies from the last 25 years investigating indications for IOL exchange and explantation reveal the changes that have occured over the last few decades (Table 6). In a study evaluating patients from the 1990s, a period of rapid development in lens technology and surgical techniques, the most common indication for IOL exchange or removal was bullous keratopathy.^3^ In light of advancements in microsurgery, 2 studies evaluating IOL exchange surgeries performed in the 2000s reported the most frequent indications as refractive errors and IOL opacification.^4,5^ Jason et al.^1^ found IOL decentration as the most frequent indication in their study. Mamalis et al.^6^ reported that IOL dislocation was the most common cause of IOL exchange or removal after cataract surgery, regardless of IOL type. In a study by Jin et al.^4^ evaluating cases between 1998 and 2004, the most common indication for IOL exchange was incorrect IOL power calculation (41%), whereas in a similar study Lyle and Jin.^3^ the most common indication for IOL removal was corneal decompensation (38%). In the present study, we found IOL subluxation to be the most common reason for IOL removal or exchange.

Jason et al.^1^ evaluated patients who underwent IOL exchange between 2007-2011 and reported that IOL dislocation was the indication in 45% of the surgeries. Similarly, we found IOL subluxation as the most common indication for lens removal in our study (58%).

Unlike dislocation of the IOL, dislocation of the lens-capsule complex can occur years after an uncomplicated surgery due to progressive separation of the zonules associated with various causes such as PEX, retinitis pigmentosa, and long axial length.^7^ In their multi-center study, Pueringer et al.^8^ analyzed nearly 15,000 cases who underwent cataract surgery within a period of about 30 years to evaluate the risk of late IOL dislocation. They reported the risk of IOL dislocation as 0.1% at 10 years and 1.7% at 25 years. Davis et al.^9^ evaluated cases with spontaneous IOL dislocation who underwent IOL repositioning and reported that independent of lens type, the presence of PEX was the most important risk factor for lens dislocation, followed by previous vitreoretinal surgery and trauma. In our study, PEX was the most common ocular pathology in patients undergoing IOL exchange.

Jason et al.^1^ reported that in PEX patients with dislocated IOL, 40% had an open posterior capsule, posterior capsule was opened in 10% with YAG laser capsulotomy, and the others had intact posterior capsules. In another study, PEX patients with IOL subluxation underwent IOL exchange or repositioning with good visual prognosis and a very low rate of intra- and postoperative complications.^2^

Jin et al.^4^ reported that a visual acuity better than 20/40 was achieved in 90% of patients who received an anterior chamber IOL. With technological advancements, open-loop, flexible anterior chamber IOLs have become a lens replacement option that provides good visual outcomes. In a study by Kwang et al.^10^ comparing anterior chamber and scleral-fixated IOLs in patients with inadequate posterior capsule support, scleral-fixated IOLs were associated with lower rates of intra- and postoperative complications, while anterior chamber IOLs resulted in better final visual acuity outcomes. Erçalık et al.^11^ compared the clinical outcomes of anterior chamber and scleral-fixated secondary IOLs after complicated phacoemulsification surgery with 15 cases in each group and reported comparable results. However, in the present study, the youngest patient in the anterior chamber group was 67 years old. Marques et al.^12^ reported secondary inflammation as the most common indication for the explantation of anterior chamber IOLs. Of the 14 patients in this study who remained aphakic, 7 had anterior chamber IOLs and 7 developed bullous keratopathy. However, despite 4 of these patients being under 50 years old (two were under 40 and 1 was under 30), they received anterior chamber IOLs after cataract removal in their initial surgery. Regardless of the advances in IOL technology in the last few decades, anterior chamber IOLs inevitably increase endothelial cell loss. We believe that the development of bullous keratopathy in these patients provides further evidence that anterior chamber IOLs are not a good choice for young patients. In our clinic, anterior chamber IOLs are not implanted in patients less than 60 years old as a principle.

In this study, 29.1% of patients with PEX were left aphakic after IOL removal, anterior chamber IOLs were implanted in 33%, in the sulcus in 15% and 15% received scleral fixated secondary IOLs. In-the-bag IOL implantation was not performed in any of the PEX patients in the second surgery. In the presence of exfoliation, spontaneous partial or complete zonular dialysis may occur after cataract surgery. In particular, dislocations occurring within the first 3 months have been associated with inappropriate capsulorhexis, while dislocations occurring after the first 3 months have been associated with zonular weakness.^9^ This explains why IOLs could not be implanted in the capsular bags of PEX patients during their secondary surgery for IOL exchange. Shingleton et al.^2^ looked at the cases of PEX patients with early and late period IOL dislocation after cataract surgery and found that the IOL was exchanged in 85% and repositioned in 15% of the cases. However, their study included patients whose only indication for repeated surgery was IOL dislocation; patients undergoing IOL removal due to bullous keratopathy were not included as they were in our study. In the current study, secondary corneal decompensation was the primary reason for not replacing the explanted IOL, and was also the second most common indication for IOL removal.

Of the 7 patients in our study who were left aphakic after IOL explantation due to corneal decompensation and edema, 3 were scheduled for keratoplasty, while the other 4 patients were unwilling to face the risks associated with keratoplasty because of their advanced age and good vision in the fellow eye. Duran et al.^13^ evaluated the indications for and outcomes of 29 cases undergoing anterior chamber IOL explantation and reported that corneal decompensation was the indication for IOL explantation in 22 cases. Three of those underwent keratoplasty and scleral-fixated IOL implantation; no surgical intervention was performed in the remaining cases.

## CONCLUSION

IOLs may require surgical correction due to complications of previous cataract surgery, late IOL subluxation, or inaccurate biometry. This risk is higher in patients with factors like PEX, previous vitreoretinal surgery and trauma. The decision of whether to replace the IOL and in which anatomic position to implant the replacement IOL depends on the patient’s anterior and posterior segment examinations, age, general health, and the surgeon’s experience and preference.

### Ethics

Ethics Committee Approval: Approval for the study was granted by the Ankara Numune Training and Research Hospital Ethics Committee, Informed Consent: It was taken.

Peer-review: Externally peer-reviewed.

## Figures and Tables

**Table 1:**
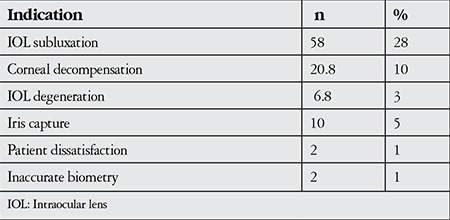
Patients’ indications for intraocular lens exchange in a second surgey

**Table 2:**
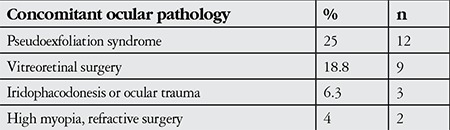
Patients’ concomitant ocular pathologies

**Table 3:**
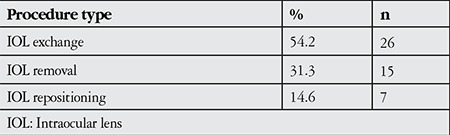
Final surgical procedure type

**Table 4:**
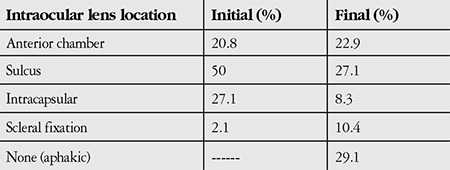
Initial and final intraocular lens positions

**Table 5:**
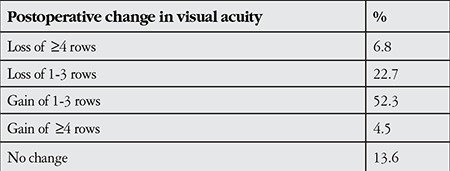
Change between visual acuity after first surgery and final visual acuity

**Table 6:**
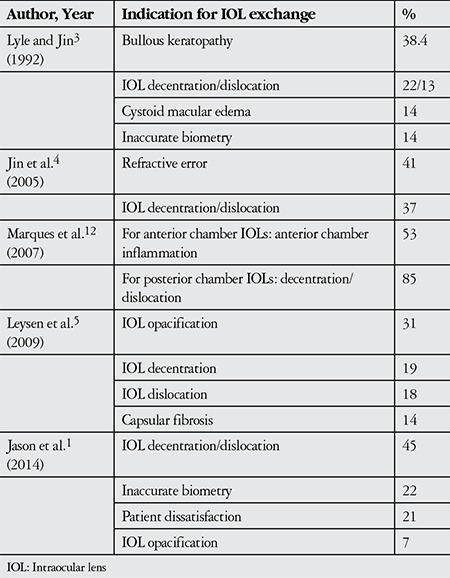
Summary of the indications for intraocular lens exchange in the last 25 years of literature
